# Daily microbial rhythms of the surface ocean interrupted by the new moon—a lipidomic study

**DOI:** 10.1093/ismeco/ycaf044

**Published:** 2025-03-09

**Authors:** Jiwoon Hwang, Alexander Hayward, Laura E Sofen, Kathleen J Pitz, Francisco P Chavez, Bethanie R Edwards

**Affiliations:** Department of Earth and Planetary Science, University of California - Berkeley, Berkeley, CA 94720, United States; National Centre for Climate Research (NCKF), Danish Meteorological Institute, Copenhagen, DK-2100 Copenhagen Ø, Denmark; Bigelow Laboratory for Ocean Sciences, East Boothbay, ME 04544, United States; Monterey Bay Aquarium Research Institute, Moss Landing, CA 95039, United States; Monterey Bay Aquarium Research Institute, Moss Landing, CA 95039, United States; Department of Earth and Planetary Science, University of California - Berkeley, Berkeley, CA 94720, United States

**Keywords:** lipidomics, diel oscillation, new moon, intact membrane lipids, phytoplankton pigments, microbial ecology, triglycerides, ocean biogeochemistry, tidal forcing, ocean carbon cycle

## Abstract

Lipids are essential biomolecules for cell physiology and are commonly used as biomarkers to elucidate biogeochemical processes over a large range of environments and timescales. Here, we use high-temporal-resolution lipidomic analysis to characterize the surface ocean community in the productive upwelling region overlying the Monterey Bay Canyon. We observed a strong diel signal with a drawdown of lipids at night and an increase during the day that seemed to correspond to wholesale removal of lipids from the surface ocean as opposed to internal metabolism. Individual lipid species were organized into coregulated groups that were interpreted as representing different phytoplankton guilds. Concentrations of long-chained triacylglycerols (TAGs) showed unique patterns over the course of five days. TAGs were used to estimate the amount of energy cycled through the surface ocean. These calculations revealed diurnal carbon cycling that was on scales comparable to net primary production. The diel pattern dissipated from most lipid modules on Day 3 as tidal forcing increased at our site with the advent of the new moon. Pigment analysis indicated that the community shifted from a diatom-dominated community to a more diverse assemblage, including more haptophytes, chlorophytes, and *Synechococcus* during the new moon. The shift in community appears to promote higher nutritional quality of biomass, with more essential fatty acids in the surface ocean during the spring tide. This analysis showcases the utility of lipidomics in characterizing community dynamics and underscores the importance of considering both diel and tidal timescales when sampling in productive coastal regions.

## Introduction

Continental shelves are highly productive regions that play an important role in global biogeochemistry, accounting for ~50% of carbon export to the deep ocean [1] as well as having large ecological impacts, supporting 95% of the world’s fisheries [2]. However, these coastal regions are physically dynamic environments with complex bathymetry, coastal upwelling, and ever-oscillating tidal cycles. Microbial ecosystems in these nearshore zones are constantly adapting to unpredictable niche changes in the waters they inhabit [3–5].

Through long-standing coastal time series such as the San Pedro Ocean Time-series, Monterey Bay Time Series, Monitoring Oregon Coastal Harmful Algae, and Ocean Station Papa, we have gained decadal and interannual insights into these systems [6–9]. Process studies, as well as autonomous deployments [10, 11], have begun to tease apart even shorter timescale variability, such as the multispecies response of the planktonic community to diel forcing, nutrient availability, and top–down control from viruses [10, 12]. Diel transcriptomic studies in the field have also observed oscillations in gene expression across ecosystems, metabolic classes, and taxa [11–14]. As would be expected, gene expression by phototrophs is tightly linked to light availability over the day. However, many heterotrophic metabolisms and viral gene expression also exhibit diel periodicity [10–14]. Phytoplankton vertical migration by motile species and diel vertical migration by grazers are well-known phenomena thought to significantly impact biogeochemical cycling in the ocean [15–17].

Diel meta-omics studies in the oligotrophic open ocean at Station ALOHA (A Long-term Oligotrophic Habitat Assessment) reveal the internal metabolism of TAGS, pigments, and osmolytes by phytoplankton as the processes that dominate the metabolomic signal in such low-nutrient environments [18–20]. The oligotrophic meta-omics studies also indicated a temporal mismatch between when a gene is expressed and the associated response of the metabolite pool [18–21]. This showcases the role of the metabolome as a comprehensive understanding of chemical transformations, reaction rates, and fluxes, which transcriptomics alone cannot capture. However, there is a lack of diel metabolomic studies in coastal regions, where processes such as tides and hotspots of grazing via zooplankton [22] significantly influence diel patterns in the surface community. The wider biogeochemical and ecological implications of such coordinated metabolic and behavioral responses necessitate a molecular-level understanding of these intricate mechanisms.

Lipids make up the more hydrophobic component of the metabolome. Lipids themselves have long been used as biomarkers of various biological processes such as photosynthesis [23] and stress response [24], as well as serving as important chemotaxonomic markers for identifying different microorganisms [25–29]. With advancements in mass spectrometric technology, the comprehensive analysis of nearly the entire lipidome has become possible, allowing for a more holistic understanding of marine microbial processes [18, 19, 25, 27, 30–36].

Here, we present a diel lipidomic time series collected over 5 days at the Monterey Accelerated Research System (MARS) station in the California coastal ecosystem (CCE). The objectives of this study were to explore lipid cycling in the coastal surface ocean over the day, the community ecology over several days, and the carbon flow through a productive upwelling system. We observed strong diel patterns at the beginning of our time series that were complicated by increasing tidal influence toward the end of our time series, which was concomitant with a shift in the phytoplankton community.

## Materials and methods

### Experimental design

Samples were collected at 12-h intervals at midnight and noon (local time) using Niskin bottles attached to a Conductivity-Temperature-Depth (CTD) rosette with a fluorescence sensor to profile oceanographic measurements and the chlorophyll *a* concentration from the surface to 200 m. Only surface (~0.5 m) samples were analyzed in this study. For particulate lipid analysis, 1 l of seawater was filtered onto a 0.2 μm Durapore filter, which was immediately flash-frozen in liquid nitrogen and stored at −80°C.

### Lipid extraction & analysis

Lipids were extracted using a modified Bligh and Dyer technique [31] and EquiSplash LIPIDOMIX® Quantitative Mass Spec Internal Standard (Avanti Polar Lipids, Alabaster, AL, USA) was added to each extraction as an internal standard. Extracted lipids were analyzed using reverse-phase Ultra-high performance liquid chromatography- mass spec/mass spec (UPLC-MS/MS) on a Vanquish UPLC with an Accucore C8 column (155 mm × 2.1 mm × 2.6 μm) in tandem with an Orbitrap ID-X mass spectrometer (all from Thermo Scientific, San Jose, CA, USA) [18]. Due to known issues with ion suppression, TAGs were analyzed using a modified method (SI text), with a lower flow rate, longer isocratic hold, and MS^3^ trigger method [37]. A chemoinformatic pipeline (Fig. S1) was used for feature detection that employed eXtensible Computational Mass Spectrometry (XCMS). Compute And Map Extracted Relevant Analytes (CAMERA) and putative lipid annotations were assigned using the Lipid and Oxylipin Biomarker Screening Through Adduct Hierarchy Sequences (LOBSTAHS) database, which is based on MS^1^ spectra and adduct hierarchy [38]. Peak alignment and quality were verified in Metabolomic Analysis and Visualization Engine (MAVEN). Putative annotations were verified, and peaks with multiple annotations were deconvoluted with the *in silico* lipid fragmentation database in MS-Dial [39]. The peak area of each feature was normalized to the recovery of the internal standard, and the procedural blank was subtracted from all samples and compounds. Absolute lipid quantification was calculated using 5-point standard curves for a suite of reference standards (see SI).

### Chemotaxonomic analysis

​​To determine the biomass of phytoplankton groups, we employed the *phytoclass* software outlined by Hayward *et al*. [29]. Analysis was run using concentrations of measured pigment lipids (peridinin, fucoxanthin, prasinoxanthin, zeaxanthin, lutein, 19′-hexanoyloxyfucoxanthin, 19′-butanoyloxyfucoxanthin, chlorophyll *a* and *b*) quantified as ng/l (Fig. S2). Prior to inverting phytoplankton pigments into the biomass of phytoplankton groups, pigment samples first underwent cluster analysis to ensure homogeneity and low variance between pigment samples. First, pigments with zero mass were imputed to 0.1% of their maximum concentration; pigments were then converted to ratios of total chlorophyll *a* biomass. To ensure normality, pigment samples were then transformed using the Box–Cox method [40]. Pigment concentrations from each cluster were then processed using the *phytoclass* simulated annealing algorithm. A step of 0.008 was used over a total of 700 iterations. The following phytoplankton classes were selected for our analysis: diatoms, haptophytes, green algae, dinoflagellates, pelagophytes, and *Synechococcus.*

### Statistical analysis

Statistical analysis was carried out in the web-based metabolomics analysis platform MetaboAnalyst (v 5.0; [41, 42]). Peak intensities were mean-centered and base10 log-transformed to scale compounds of varying absolute intensities. Multivariate analyses ([Orthogonal] Projections to Latent Structures Discriminant Analysis [OPLS-DA, PLS-DA], Principal Component Analysis [PCA]) were supported by ancillary Random Forest classification methods [43]. Multivariate analyses were subjected to cross-validation (Figs S3 and S4) to evaluate for data overfitting, which is known to accompany such analyses when the number of samples is much smaller than the number of variables for each sample.

The Weighted Gene Correlation Network Analysis (WGCNA) R package (v 1.70; [44, 45]) was used to perform hierarchical clustering on the relative abundance of the intact polar lipids and TAGs within the meta-lipidome, following the adaptations made for metabolomics data from Pei *et al*. [46]. A signed soft thresholding power of 24 was used to obtain a sufficiently high scale-free topology (*R*^2^ ≈ 0.75) while not compromising all the mean connectivity. Compounds that did not cluster into any module were assigned to a separate group (grey module) that was not counted as an individual module.

### Absolute quantification of the change in triacylglycerol-associated carbon over time

Absolute values of TAG-associated carbon in the surface ocean were approximated by converting the peak intensity of each TAG compound to moles per liter based on the standard curve (Fig. S5), multiplying by the number of carbons in that molecule and then converting moles of carbon per liter to grams of carbon per liter. Daily nocturnal losses were calculated by subtracting the nighttime values from the preceding daytime values, and daily production was calculated by subtracting the daytime values from the preceding nighttime values.

TAG measurements underwent particularly rigorous quality control. In addition to a modified analytical method, TAGs were manually verified for signal-to-noise ratio and proper peak integration via an XCMS–CAMERA–LOBSTAHs pipeline paired with MAVEN. Peak areas of poor XCMS peaks were retrieved via MAVEN and corrected by multiplying the average observed ratio between the MAVEN and XCMS output of verified peaks.

## Results

### Temporal patterns observed in environmental parameters

The waters overlying the continental slope that lies north of Monterey Canyon are highly dynamic, with turbulent coastal mixing complicated by the steep bathymetry of Monterey Bay (Fig. S6) and intricate tidal flow patterns. Such temporal and spatial dynamism was evident in a range of environmental parameters measured over our 5-day sampling period. Samples are hereby denoted as day (D) or night (N) accompanied by the number of the sampled day.

**Figure 1:**
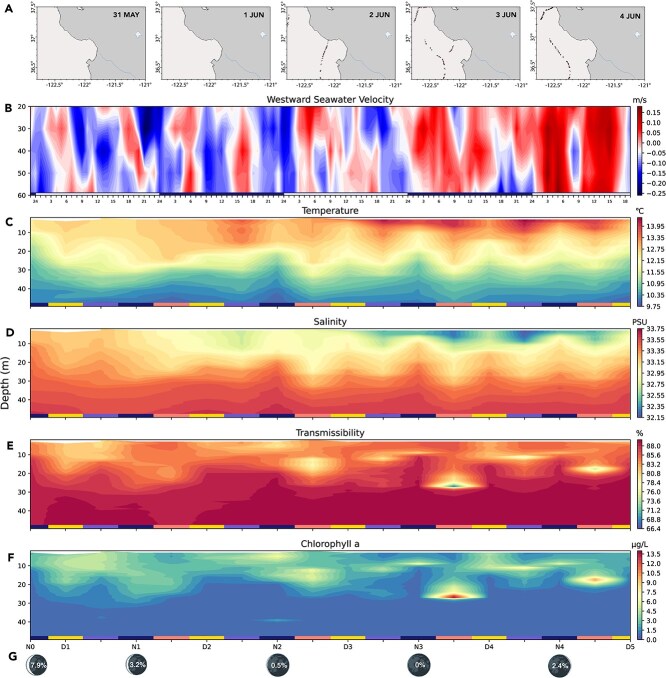
Physical oceanographic parameters during the sampling period. (A) Frontal edge formation from 31 May (D1) to 4 June 2019 (D5) (NOAA ERDDAP). (B) Westward seawater velocity (Mooring 1, CeNCOOS). Alternating white–dark bars indicate days. (C–F) PCTD measurements of temperature, salinity, transmissibility, and chlorophyll *a*. Alternating colored bars on the x-axis indicate midnight (0:00), dawn (6:00), noon (12:00), and evening (18:00) casts. While CTD measurements were carried out every 6 h, only midnight and noon casts were sampled for the particulate lipidome. These casts are marked on the *x*-axis as N0 for the first night (0:00) cast, D1 for the first day (12:00) cast, and so on. (G) Moon phases marked with percentage of illumination.

For the initial three days of this time series (from N0 to D3), oceanographic variables of the surface ocean such as temperature and salinity (Fig. 1C and D) were relatively stable over time. However, during the latter duration of sampling (from N3 to D5), a body of warmer and less saline near-shore seawater was brought out to the surface waters of the MARS station. PCA of temporal differences between successive measurements of surface (~0.5 m) temperature, salinity, and oxygen concentrations (Fig. S7A) separated the data points into two largely distinct clusters that represented the first and second half of the data collection period.

The sharp increase in the magnitude of the semidiurnal fluctuations in physiochemical parameters (Fig. 1B–F) at MARS after D3 coincided with the new moon rising on N4 (Fig. 1G) and the resulting spring tide. Although tidal intensity does not increase abruptly, sudden changes in oceanographic conditions can accompany the movement of sharp spatial gradients in the water column, also known as fronts. Local satellite data show that a front was formed between D2 and D3 and moved out to sea on D4 and D5 (Fig. 1A). This front was transported out to our site by the strong westward flow of seawater on D4 (Fig. 1B) that accompanied the spring tide.

### Particulate lipidome analysis shows distinct temporal trends

**Figure 2:**
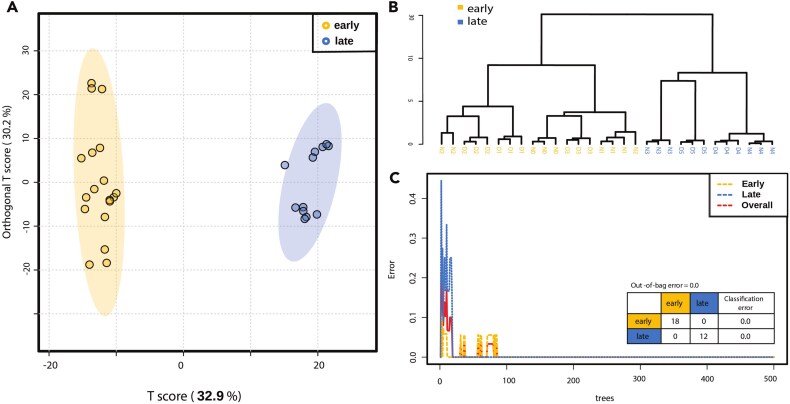
Multivariate and machine learning–based analyses of samples (*n* = 30) categorized into early (*n* = 18)/late (*n* = 12) sampling phases. (A) OPLS-DA score plot. Shading indicates the 95% confidence interval. (B) Dendrogram using a Pearson correlation matrix and Ward clustering. (C) Random Forest classification results and out-of-bag error. Cross-validation results can be found in the Supplemental Material (Figs S3 and S4).

Both multivariate analysis (PCA, OPLS-DA) and machine learning–based (Random Forest) statistical analyses on the particulate lipidome of the surface ocean (0.5 m) pulled the sampling periods of N0–D3 (*n* = 18) and N3–D5 (*n* = 12) apart (Fig. 2), agreeing with the two distinct clusters identified in the PCA of oceanographic parameters (Fig. S6A). The lipid classes represented in this analysis included pigment lipids, neutral storage lipids such as TAGs, and intact polar glycerol lipids that constitute the cellular membrane, such as phospholipids, glycolipids, and betaine lipids (Table 1). These analyses also revealed a strong diel pattern in the surface ocean lipid pool early on in our time series, with a significant distinction between day and night lipidomes for all lipid classes during this early period (Table S1). The diel signal was dampened during the latter segment of the time series, as shown by the low OPLS-DA T-score for the day–night lipidomes, compared to that of the early period.

**Table 1:** Intact polar lipid classes and their chemotaxonomic properties in the oxic surface ocean.

Lipid class	General characteristics	Species	Species-specific properties	Reference
Glycolipids	Chloroplast lipids; thylakoid membranes of phytoplankton including cyanobacteria	Monogalactosyldiacylglycerol (MGDG)	Thylakoid membranes of photosynthetic organisms, heterotrophic bacteria	[27, 47]
		Digalactosyldiacylglycerol (DGDG)	Thylakoid membranes of photosynthetic organisms	[27]
		Sulfoquinovosyldiacylglycerol (SQDG)	Mainly produced by photosynthetic surface organisms as a thylakoid membrane lipid	[27, 48]
Phospholipids	Head group contains phosphorus; PC and PE also contain nitrogen; PDPT also contains sulfur	Phosphatidylethanolamine (PE)	Major component of heterotrophic bacteria	[32, 48–50]
		Phosphatidylcholine (PC)	Eukaryotes such as brown algae, heterotrophic bacteria. Not found in cyanobacteria	[26, 27, 31, 32, 50]
		Phosphatidylglycerol (PG)	Heterotrophic bacteria; also linked to cyanobacteria as a minor component of the thylakoid membrane	[27]
		Phosphatidyldimethylpropanethiol (PDPT)	Biomarker for *Emiliania huxleyi*	[51]
Betaine lipids	Head group contains nitrogen. Specific to “photosynthetic eukaryotes” in the oxic water column but can be produced by bacteria under anoxic conditions. Produced in the endoplasmic reticulum (ER)	Diacylglyceryl trimethylhomoserine (DGTS)/Diacylglyceryl hydroxymethyl-trimethyl-β-alanine (DGTA)	DGTS—green algae and bacteria under P-limited conditions DGTA—brown algae	[26, 50, 52]
		Diacylglyceryl carboxyhydroxymethylcholine (DGCC)	Mainly associated with haptophytes and dinoflagellates although also produced in low concentrations by diatoms and chlorophytes	[26, 50, 53–55]
		Betaine-like lipid (BLL)	First isolated in *E. huxleyi*	[51]

### Pigment biomarkers point to temporal changes in community

**Figure 3:**
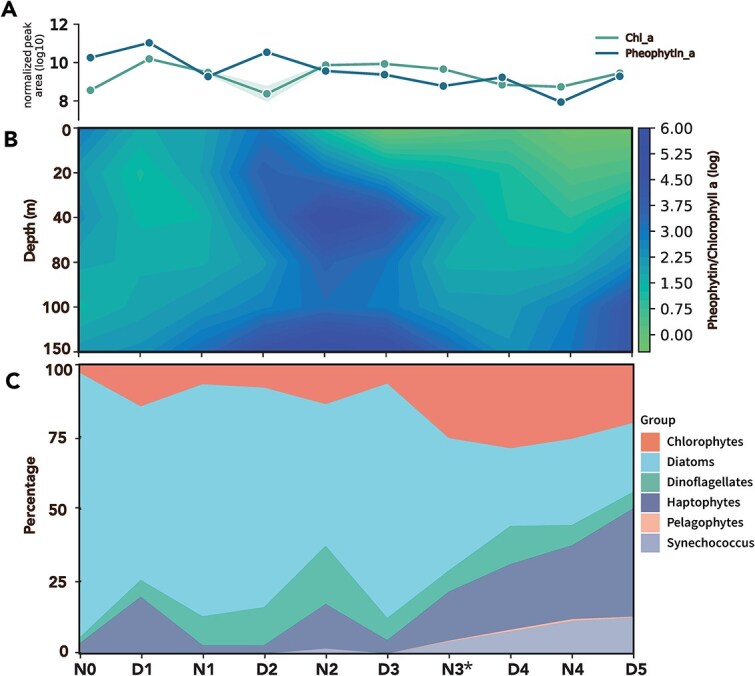
Pigment-based analyses over time and depth. (A) Pheophytin *a* and chlorophyll *a* at 0.5 m, peak areas log_10_-transformed (B) pheophytin *a:* chlorophyll *a* over 0.5–150 m, peak areas log-transformed. (C) Pigment-based *phytoclass* analysis results; see Table S2 for standard deviation. Asterisk (*) indicates the new moon on N3.

Chemotaxonomic analysis based on the *phytoclass* algorithm [29] with nine types of pigment lipids demonstrated a shift in community structure with time (Fig. 3C, Table S2). We observed low diversity and very high diatom dominance, comprising up to 91% of the community structure during the beginning of the time series. Toward the end of the time series, the phytoplankton community was more diverse with a decline in the relative abundance of both diatoms and photosynthetic dinoflagellates, whereas the relative abundances of green algae, haptophytes, and *Synechococcus* increased. There was little signal from pelagophytes throughout the analysis.

We used the pheophytin *a*: chlorophyll *a* ratio as a proxy for general bloom demise, with healthy cells assumed to have a standard ratio of 3:1 [19]. This proxy indicated an initial community with declining physiological cell state succeeded by a healthier community (Fig. 3A and B). Combined, the physicochemical parameters and pigment-based chemotaxonomic methods showed the replacement of a declining diatom bloom with a healthy and more diverse community. This community change was concomitant with the new moon on N3 (Fig. 1G) and increased offshore currents (Fig. 1B) with a decrease in the wind-driven upwelling on D2–D4 (Table S3).

In addition to their chemotaxonomic and light-harvesting properties, pigment lipids also serve as photoprotective compounds. For example, diatoxanthin and zeaxanthin are both the de-epoxidized xanthophylls of their respective nonphotochemical quenching xanthophyll cycles. When normalized to biomass, these pigments increased during the latter part of the time series (Fig. S8B), when there was higher optical clarity in the surface ocean (Fig. 1E). Biomass-normalized values of the red antioxidant carotenoid, astaxanthin, also increased during this period.

### Network analysis of glycerol lipids showed distinct functional modules

**Figure 4:**
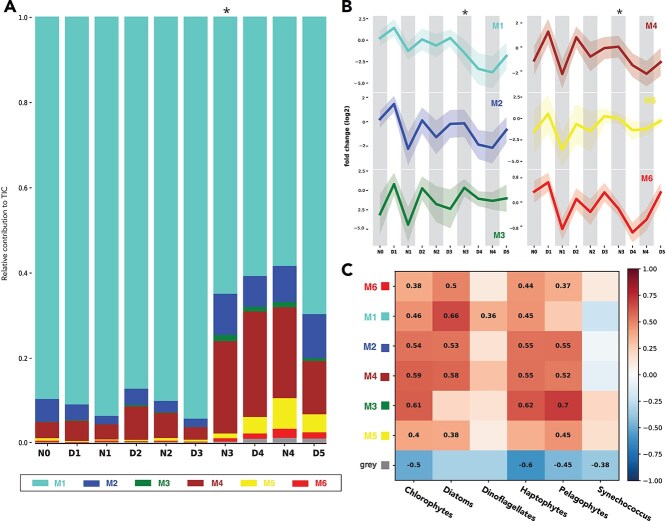
WGCNA of intact polar lipids and TAGs (n = 658) including betaine lipids (n = 69), thylakoid membrane lipids (n = 70), and phospholipids (n = 108) as well as neutral storage lipids such as triglycerides (n = 150) and wax esters (n = 29). Detailed breakdown of lipid species can be found in Fig. S10. The gray module was not included in our overall analysis, due to its status as the “nonmodule module” where compounds that are not significantly correlated with an expression module are placed. (A) Relative module contribution to the total ion chromatogram (TIC), averaged by the number of compounds in each module (B) Average 2-fold change over time; the y-axis is the normalized, mean-centered, and log2-transformed peak area, shading = 1σ across all compounds in module. (C) Pearson correlation between WGCNA modules and phytoplankton subgroups. Only correlations with *P*-values <.05 are marked. Asterisks (*) above graphs indicate the new moon on N3.

The 652 lipid compounds annotated as free fatty acids, wax esters, stanol and sterol esters, and glycerol lipids, namely, intact polar lipids (IPLs) and TAGs, were analyzed using WGCNA, an R-based package that uses network analysis to look for similar concentration patterns to identify coregulated groups (“modules”) of compounds. Among the six total modules classified by WGCNA (the clustering diagram is available in the supplementary GitHub repository), five modules (M1–4, 6) were made up of an array of lipid species including IPLs (Fig. 4), while the other module (M5) consisted exclusively of TAGs (Fig. 4; navy circles) The fact there is a TAG-exclusive module underscores the distinct role of TAGs as the main energy storage molecules of the surface community, which is markedly different from the structural functions of the membrane lipids.

**Figure 5:**
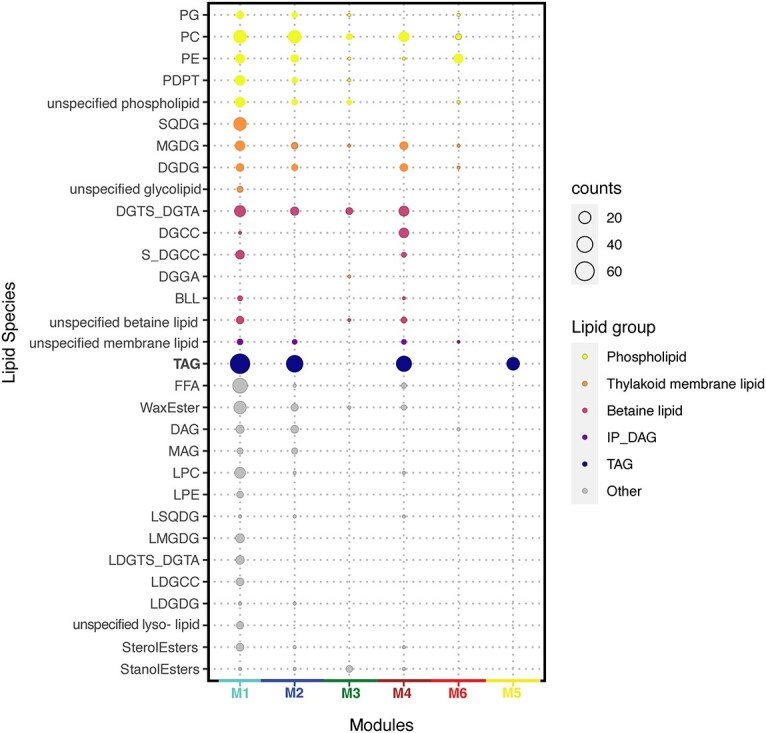
Lipid compositions of each WGCNA module. Radii of bubbles correspond to the number of lipid compounds within that category. Colors of module names on the x-axis correspond to module colors in Fig. 5. Specific lipid classes are presented in detail in Fig. S9. Module compositions of lipid classes are presented in Fig. S10. “Unspecified” prefix indicates co-annotation between lipid species within the same subclass.

Two types of chloroplast lipids, monogalactosyldiacylglycerol (MGDG) and digalactosyldiacylglycerol (DGDG), along with one type of betaine lipid, diacylglyceryl trimethylhomoserine/diacylglyceryl hydroxymethyl-trimethyl-β-alanine, which are associated with photosynthetic organisms (Table 1), were always present in at least four of the IPL-containing modules (Fig. 4, S9, and S10; full abbreviations shown in Table 1), while two types of phospholipid, PC and PE, were present in all five. These modules exhibited a day-high, night-low pattern during the beginning of sampling that disappeared during the latter portion (Fig. 5B), as was alluded to in the OPLS-DA. Interestingly, sulfoquinovosyldiacylglycerol (SQDG) compounds were only found in M1, while DGCC was mostly found in M4 (Figs 4 and S10). The division between modules was mostly influenced by distinct changes in the latter half of the sampling period. We hypothesize that these differences arise from the diverse functional guilds of species that are represented in each module and will characterize these guilds in this section.

M1 was the largest module, with *n* = 348 lipid species, and the most diverse; 25 of all 26 lipid subclasses were represented (turquoise; Figs 4 and S9). In addition to the more general phytoplankton indicators, this module all 26 annotated SQDGs (Figs 4 and S10), which are known to be mainly produced by photosynthetic surface organisms (Table 1). This module also contained the highest number of TAGs (Fig. 4). Therefore, this module is likely to be associated with TAG-heavy photosynthetic surface organisms, such as diatoms. This is supported by module–trait correlation analysis of *phytoclass*-based species abundances and WGCNA modules (turquoise; Fig. 5C) that showed that M1 had the highest correlation with diatoms (Pearson correlation index = 0.66, *P* = .00007). This module was characterized by an acute decrease in concentration during the latter part of the sampling period (Fig. 5B).

M2 (blue) showed temporal trends similar to M4 (brown; Fig. 5) but was compositionally distinct (Fig. S9). M2 was dominated by phospholipids (52% of all non-TAG M2 lipids) whereas M4 was mostly dominated by betaine lipids (44% of all non-TAG M4 lipids) DGCC was found almost exclusively within M4 (92.3%; Fig. 4) and characterized this module as a haptophyte-related guild (Table 1). This guild assignment is supported by the increased contribution of this module to the total ion chromatogram during the latter sampling period (Fig. 5A), which agrees with the relative rise of haptophytes during this period (Fig. 3C).

M3 (green) is the only IPL module that did not exhibit a substantial decrease in fold change during the latter sampling period (Fig. 5B). It also included the majority (58%) of the stanol esters, which are major constituents of dinoflagellates and *Pavlova* spp. but are not commonly found in other microalgal species [56]. M3 also shows a high correlation to haptophytes, chlorophytes, and pelagophytes (Fig. 5C).

M6 (red) was almost entirely dominated by phospholipids (80%), particularly the PEs (Figs 4, S9, and S10). Therefore, this guild appears to be associated with heterotrophic bacteria (Table 1), as is corroborated by the low correlation to any of the photosynthetic species identified by *phytoclass* (Fig. 5C). M6 was also characterized by an acute decrease during D5 (Fig. 5B).

M5 (yellow) consisted entirely of TAG compounds (Fig. 4) and appeared to increase relatively over time (Fig. 5). The mean number of total fatty acid chain carbons was the highest in M5 with 10 more carbons on average (56 carbons per molecule; Fig. S11) compared to the overall average for all TAGs in the observed lipidome (47.5 carbons per molecule; M1–2, 4–5), as well as being more unsaturated (9.2 double bonds per molecule compared to 6.9 overall).

## Discussion

### Physiochemical measurements show two temporally distinct water masses

Overall, this lipidomic dataset was strongly characterized by clear diel periodicity during the early period of low coastal forcing, which was subsequently disrupted by the movement of a front that appeared after the new moon (Figs 1, 2, and 5B). The MARS station is very close to the shelf break, where the continental shelf drops off into deeper oceanic waters (Fig. S6). When tidal waves move across this topology, they shallow rapidly. This abrupt physical change creates an environment conducive to the formation of tidal fronts [4, 57–59]. Such tidal fronts in the southeastern English Channel have been shown to separate water masses with distinct oceanographic features such as primary production, community composition, and nutrient concentration [60].

The physiochemical parameters suggest that the strong physical dynamics that brought coastal freshwater further out to sea during the spring tide caused substantial interference with the observed diel signal. This contrariety was compounded by a temporal mismatch between the frequency of the two periodic phenomena (the 24.83-h lunar day and 24-h solar day). We also noted that the discontinuity of the sampled community in this station-based sampling scheme may have contributed to this disruption. Together, these two factors were the most likely reasons for the apparent weakening of the diel signal. In addition, the 12-h sampling frequency employed in this study provided a broader assessment of daily trends and may not have captured the full diurnal range of the lipidome. Future Lagrangian sampling schemes in coastal regions that sample based on the 24-h daily cycle will simultaneously integrate the cycle of the high–low tide and may therefore help pull apart the multitude of dynamics influencing coastal systems. Henceforth, we considered these two periods as separate regimes being influenced by distinct environmental forcings and discussed them as such. The “early” period (N0–D3) can be described as an oceanic regime with a periodicity that is mostly governed by the daily solar cycle, while the “late” period (N3–D5) is dominated by coastal influence with the tides dictating the temporal rhythm.

### Diel pattern in the early-phase lipidome

The distinct day-high, night-low pattern that was typical of the early phase of this lipidome was strongly represented in both the thylakoid glycolipids and betaine lipids (Fig. S12). Here, we considered the thylakoid membrane lipids (MGDG, DGDG, SQDG; Table 1) as a proxy for prokaryotic and eukaryotic algal biomass as a whole. We also considered the betaine lipid DGCC, as it is known to have a positive linear relationship with eukaryotic algal cell counts and has been used as such in previous lipidomics studies [33, 61]. Both biomarkers indicated a wholesale removal or transport of photosynthetic organisms at night followed by photosynthetic production or transport to the surface during the daytime.

### Nocturnal biomass removal processes and implications

Nighttime removal during this early period was consistent with nocturnal patterns of grazing or viral lysis, as diel vertical migration of micro- and meso-zooplankton is well documented in Monterey Bay [62], while viral infection in the coastal oceans has also been shown to exhibit diel periodicity [12, 63, 64]. To gauge the amount of externally consumed carbon moving through the surface ocean of this station we calculated the change in carbon content associated with the carbon-dense and energy-rich storage lipids, TAGs (Table 2 and Figure S6). Thus, we were able to assess the flow of calorific energy through the ecosystem by means of a lipid chemical currency, TAGs, which are known to be the main mediums of energy storage, consumption, and generation in the cell.

**Table 2:** Measurements of NPP and back-of-the-envelope calculations of biomass based on molar mass and carbon content of quantified lipids.

	Units	Sampling day 2	Sampling day 3	Sampling day 4	Sampling day 5
Surface NPP [65]	mgC/m^3^/day	261	171.7	127.8	73.6
Euphotic zone NPP^a^ (depth)	mgC/m^2^/day	2151.2 (30.1 m)	3425.7 (62.3 m)	1357.8 (45.4 m)	1303.6 (41.7 m)
TAG loss (σ)	mgC/m^3^/day	149.6 (36.8)	32.8 (17.8)	11.1 (7.3)	−4.2 (3.5)

Values are for 0.5 m and not integrated over depth unless otherwise stated.

a Calculated by integrating measured values of PP over the euphotic zone.

Assuming TAG loss at night was entirely due to grazing, the amount of carbon nocturnally transferred to upper trophic levels (calculated as the nighttime carbon concentration subtracted from the prior daytime concentration) in the early period (N0–D3) was equal to up to 43%–57% of measured surface net primary production (Table 2). This range is a result of a methodological difference between our lipidomic analysis, which assessed the >0.2 μm fraction of particulate matter, and the NPP analysis, which filtered the >0.7 μm fraction [65]. In an iteration of the Community Earth Systems Model, pico-phytoplankton (0.2–2 μm) were found to contribute 58% to NPP [66]. While a DNA study across the North Pacific found that the 0.2–0.7 μm fraction of the CCE was dominated by chlorophytes and photosynthetic dinoflagellates [67]; both taxa taken together make up ~25% of the community composition in our study (Fig. 3C), resulting in the low-end calculation of 43%. In the oligotrophic open ocean, diel TAG cycling was 6.4 ± 1.7% of total daytime net primary production in the summer and was attributed to internal consumption based on stable DGCC concentrations [18]. However, in our coastal ocean ecosystem, wholesale biomass change dominated the diel signal in TAGs, as evidenced by a concomitant change in DGCC as well as most other lipids in the lipidome (Figs 5 and S12). Thus, we chose to ignore internal cycling in our assessment of TAG loss as it was likely a minor component in this productive region. NPP values are presented for contextual purposes only, as under our non-Lagrangian sampling scheme, nighttime grazers may not have been feeding in the same water mass where daytime production predominantly occurred. Our calculated grazing percentage was higher than the overall global budget, which is estimated at 74% of PP consumed via grazing of phytoplankton [68]. We also did not observe the same day-low, night-high pattern in photosynthetic pigments that Becker *et al*. [19] saw at St. ALOHA, further confirming different processes govern lipid cycling in the coastal ocean (wholesale removal) vs. the oligotrophic open ocean (internal cycling). All of our pigments were day high, night low except for chlorophyll *a*, which had an erratic pattern (Figs 3A and S8).

We used the classical 10% trophic transfer rate [69] to provide a general understanding of the flow of energy through our study area. We acknowledge that transfer efficiency in temperate coastal upwelling zones can vary largely between 0.3% and 34.4% [70]. We calculated that at the surface, a grazing population of 15.0 mgC/m^3^/day could be supported by consumption of the TAG pool alone. The average heterotrophic biomass for the surface ocean of the nearby M1 station during our sampling period was roughly 29–34 mg/m^3^ [71], agreeing well with the range of this calculation. Thus, during the early sampling period, the nighttime consumption of TAGs was enough to support the biomass of the upper trophic levels. Over time, the magnitude of this nighttime consumption appeared to wane, consistent with the masked diel signal during the latter part of this time series. This was likely due to the temporal mismatch in diel sampling and tidal dynamic discussed above. Therefore, we only considered TAG consumption in the early phase.

**Figure 6:**
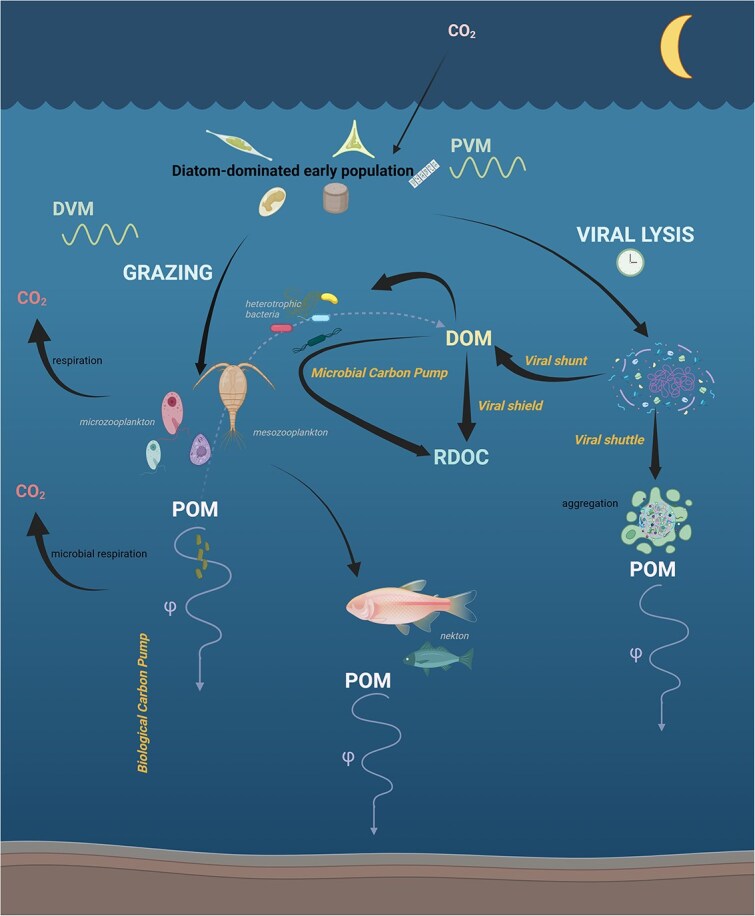
Depiction of possible nighttime surface ocean biomass removal processes and their carbon cycle trajectories. Aggregate sinking of marine snow is not represented. Removal of phytoplankton-associated biomass from the surface ocean at night could be attributed to diel vertical migration (DVM) associated with micro- and meso-zooplankton grazing, diel cycles in viral lysis, or phytoplankton vertical migration (PVM). Grazing would transfer carbon up the trophic food web to larger nekton and contribute carbon to the gravitational carbon pump via fast-sinking pellets. Viral infection would partition carbon into the dissolved organic matter (DOM) pool via the viral shunt, sinking particular organic matter (POM) pool via aggregation in the viral shuttle, or the refractory DOC pool via the viral shield/microbial carbon pump. PVM would represent physical transportation of carbon out of the surface ocean at night as phytoplankton access nutrients. Created in BioRender and modified from Zhang (2018) [85].

Nocturnal biomass removal could be partially mediated by diurnal oscillations of viral activity, which has been observed for lab-grown and *in situ* infections of cyanobacteria and picoplankton [72–74]*.* In most cases, transcription of viral genes and viral lysis were tightly coupled or synchronized with the diel periodicity of the host, peaking during the daytime*.* However, there were some notable exceptions where the transcription of the viral genome, relative to the host genome, varied based on community composition [12]*.* For example, genomes of viruses infecting *Ostreococcus* showed peak expression at night, both for in situ observations of the California Coast Ecosystem [12] and culture experiments [72]. The timing between transcription and lysis may also differ depending on the species, environmental conditions, and time of infection [72, 73]. In addition, viral lysis produces various complicated trajectories of carbon cycling that include the viral shunt, shuttle, and shield that are still largely unquantified [75]. Due to such uncertainties, we did not attempt to draw any conclusions related to viral infection in this paper. However, we acknowledge viral infection as a potentially significant player in diurnal cycles of the coastal surface community. Clearly, the fate of this disappearing carbon differs greatly depending on which mode of removal dominates the system (Fig. 6) and therefore begs further elucidation of these complex processes.

### Nighttime transport processes and implications

We also consider a scenario where biomass is not consumed but rather transported to depth at night via phytoplankton vertical migration (PVM; Fig. 6). Phytoplankton that undergo PVM move upward in the water column during the day to access sunlight for photosynthesis and descend during the night to take advantage of remineralized nutrients at depth [76]. This migratory behavior leads to higher concentrations of phytoplankton-related metabolites during the daytime and a decrease at night, consistent with the observed diel trends in our dataset. This movement of phytoplankton has previously been observed in Monterey Bay in the mixotrophic dinoflagellate *Akashiwo sanguinea* [77]; however, it is also known to occur in the mat-forming diatom *Rhizosolenia* that is found in this coastal system [76, 78]. In this scenario, we would observe the result of PVM as a nocturnal decrease in the TAG-related carbon, ergo a decrease in the calorific value of the surface ocean at night.

In summary, our assessment of carbon flux based exclusively on TAG-associated carbon (Table 2) aligned well with the range of heterotrophic biomass and parallel NPP measurements, indicating the robustness of our approach as a quantitative measure of carbon flux. Specifically, our findings demonstrate that, during periods of low tidal influence, substantial amounts of TAG-associated carbon are cycling through the surface ocean daily, on scales that are on par with levels of primary production (57%) representing essential fatty acids and high-calorie energy available to the rest of the food web. Consequently, the fate of this considerable carbon flux will significantly impact the biogeochemical processes of the surface ocean and the dynamics of carbon reservoirs. Our station-based sampling also showcases the importance of constraining the spatial distribution of microbial communities over highly variegated coastal seascapes, which is a crucial factor influencing the mode and magnitude of biogeochemical elemental cycling and carbon export (i.e. the biological carbon pump) [1].

### Long-chained triacylglycerols accumulated in the late-phase lipidome

Interestingly, the overall relative abundance of long-chained TAGs increased during the tide-dominated regime (N3–D5; Figs 5A, S9, and S13) compared to the decrease of most other compounds, especially those that are associated with phytoplankton biomass (Fig. S12). During the late period (N3–D5), TAG compounds had a relative increase, from ~30% of TIC to 45%. (Fig. S13). Approximately 35% of this increase was contributed from the TAG-exclusive M5 (52.6 μgC/l), which was predominantly made up of larger, more unsaturated TAGs (Fig. S11). High FA carbon content indicates the presence of long-chained polyunsaturated fatty acids (LC-PUFAs) such as eicosapentaenoic acid (EPA) and docosahexaenoic acid (DHA), which are crucial for intracellular signaling and metabolism regulation in vertebrates. Very few marine vertebrates produce LC-PUFAs themselves and must obtain them from their diet [79]. Hence, the accumulation of TAGs containing these essential FAs points to a surface ocean that has higher nutritional quality per unit biomass during the strong tidal period, the effects of which can reverberate up the food chain [80].

TAG accumulation is widely recognized as a stress response in eukaryotic algae to nutrient-limited and high-light environments, acting as a protective mechanism in response to reactive oxidant damage of the membrane [52]. Specifically, the production of TAG and fatty acids serves as an electron sink. For example, the synthesis of a C18 fatty acid consumes around 24 Nicotinamide Adenine Dinucleotide Phosphate Hydrogen (NADPH, critical electron carriers involved in cellular metabolism), effectively regulating the over-reduced electron transfer chain and helping to alleviate the adverse effects of excessive electron accumulation [81].

There are various arguments for this being a high-photoenergy period, such as the relative increase in photoprotective pigments (Fig. S8) and transmissometer data indicating clearer waters at this time (Fig. 1E). De-epoxidized xanthophylls such as diatoxanthin and zeaxanthin are produced to dissipate excitation energy of the photosystem under high-light conditions. As a powerful antioxidant, astaxanthin has also been proposed to be a cellular photoprotective agent via physical and chemical processes and is accumulated in response to reactive oxygen species (ROS)-producing stress conditions [82]. In addition, the surface ocean during this latter time frame was more nitrogen limited (Fig. S14) due to increased stratification of the water column (Figs 1C and D and S7B). Therefore, the increase in the relative abundance of long-chained TAGs over time suggests that the remaining diatoms and dinoflagellates are responding to the high-stress conditions of the surface ocean. Alternately, haptophytes that increase in relative abundance over time or trophic transfer could be the source of the late-period TAG signal (Fig. 3B). *Phytoclass* data indicate that long-chain TAG producers like diatoms were on the decline (Fig. 3C), so, alternatively, this increase could be caused by trophic accumulation in grazers such as heterotrophic flagellates or copepods.

### Community succession is influenced by physical forcing

Based on our chemotaxonomic analysis of pigments and IPLs, we see that the community starts as a diatom-dominated, more homogenous community that transforms into a more diverse community over time, with an increased presence of haptophytes and chlorophytes, as well as an emergence of *Synechococcus*. allowing more species to co-exist (Fig. 3). This could be the result of the removal of the monopolistic diatom community via grazing or viral lysis, as suggested in the previous section. Alternatively, a similar study conducted in the coastal Iroise Sea (France) suggested that the increase of competitive exclusion under continuous, undisturbed conditions, such as those in the early part of our time series, would decrease species diversity, while local disturbances such as those created by internal tides can lower competition, allowing more species to co-exist [5].

Similarly, physical oceanographic features and community structure are directly interconnected. Although small-scale processes such as regional-scale tidal fronts can be difficult to constrain, it has been suggested that tidal fronts could act as ecotones between oceanic and coastal phytoplankton populations, where tide-induced mixing acts as an important driver of ecological competition, community composition, and diversity [5]. Lévy *et al*. also showed that physical forcing of sub-mesoscale features is associated with phytoplankton diversity and ecosystem structure, such as physical processes that transport phytoplankton populations downward out of nutrient-rich waters or enhance their light exposure via vertical stratification and turbulence of the water column [3]. As such, lipidomic patterns that result from differing community responses can be correlated reasonably to tidal forcing, such as we have seen in this study.

In conclusion, the application of lipidomics has provided us with valuable insights into coastal ecosystem dynamics. By focusing on lipid profiles alone, we have gained a deeper understanding of the population composition, diversity, and the resulting transport of carbon within the ecosystem, which appears to be highly influenced by tidal patterns. Most lunar-oceanic studies have focused on the effects of the full moon, such as enhanced visibility and tidal strength promoting predation by visual predators and broadcast spawning events by coral and other invertebrates [83, 84]. Yet we observed that the increase in tidal forcing associated with the new moon was correlated to a change in the structure of the phytoplankton community and the nutritional quality of the surface ocean at night. These observations underscore the importance of both moon phases and concurrent tidal influences in the coastal ecosystem. We emphasize that the timescales of the spring–neap tide cycle and apogee–perigee cycle in the coastal ocean must be considered in addition to the underlying hourly diel timescales that dominate the relatively less perturbed open ocean [13, 18–21].

Our investigation also revealed diel cycling in coastal lipidomes, mostly characterized by increased levels of lipids during the day and decreased levels at night, implying the removal of lipids from the system. The weakening of these diel patterns was attributed to tidal effects. Our analysis of daily carbon flux based on TAG-associated carbon also highlights the robustness of TAG quantification in estimating carbon cycling associated with various removal mechanisms and reinforces the utility of lipids as a chemical currency for understanding the ocean’s biogeochemical processes. Our untargeted lipidomic approach to the analysis of a coastal time series has proven instrumental in revealing previously unobserved associations with the new moon and molecular, metabolic, and community-level trends within the coastal microbial ecosystem and could be paired with metagenomics or transcriptomics sequencing in future studies to provide insights into the underlying mechanisms driving these observed patterns.

## Supplementary Material

supplementary_materials_ISMEcomm_Feb2025_finalish_ycaf044

## Data and materials availability

Original data created for the study are available through the MassIVE repository (MSV000096826; doi:10.25345/C54F1MW73). Secondary data and scripts can be found on GitHub: https://github.com/jiwoon-hwang/MBTS-2023.
