# Supplementary material for: Daily microbial rhythms of the surface ocean interrupted by the new moon—a lipidomic study
Source: ISME Commun. 2025 Mar 9;5(1):ycaf044. doi: 10.1093/ismeco/ycaf044 (PMC11962720; doi:10.1093/ismeco/ycaf044)
Supplement: supplementary_materials_ISMEcomm_Feb2025_finalish_ycaf044 [file supplementary_materials_ismecomm_feb2025_finalish_ycaf044.pdf]

Supplementary Materials for

Daily microbial rhythms of the surface ocean interrupted by the new moon- a lipidomic study

Jiwoon Hwang *et al.*

*Bethanie R. Edwards. Email: bethanie_edwards@berkeley.edu

This PDF file includes:

Supplementary Text
Figures S1 to S9
Tables S1 to S3

Supplementary Text

Materials and Methods

Lipids were extracted using a modified Bligh and Dyer method following Popendorf et al., 2013. EquiSplash LIPIDOMIX® Quantitative Mass Spec Internal Standard (Avanti Polar Lipids, Alabaster, AL, USA) was diluted to 50ug/mL in methanol, and 10uL of this dilution was added to each extraction as an internal standard to correct for differences in extraction and ionization efficiency. A clean Durapore filter was extracted and analyzed as a procedural blank along with the environmental samples.

Extracted lipids were analyzed using reverse UHPLC-MS/MS on a Vanquish UHPLC with an Accucore C8 column (length 155mm, diameter 2.1mm, silica particle size 2.6um) in tandem with an Orbitrap ID-X mass spectrometer (all from Thermo Scientific, San Jose, CA, USA), using an analytical method of eluents, gradient changes, and flow rates for UHPLC as described in Becker et al., 2018 (adopted from Hummel et al 2011).

Due to known issues with ion suppression, TAGs were analyzed using a modified method, with a lower flow rate of 0.2 mL/min, and a 25-minute isocratic hold added to the end of the standard UPLC gradient to better separate TAG peaks. To distinguish between co-eluting isomeric TAGs, an ms3 trigger method was adopted from Thermo Scientific Application Note 648 (Kiyonami et al., n.d.). In brief, an ms3 scan was triggered when a neutral loss of a fatty acid plus ammonia was detected in the MS2 spectrum, enabling full characterization of the TAG molecule.

The lipidomic workflow followed can be found in Figure S1. Peaks were processed with XCMS and CAMERA (XCMS (v 3.16.1; Smith (2006), Tautenhahn (2008), Benton (2010)); CAMERA (v 1.50; Kuhl (2012)) packages in R (v 4.1.2; R Core Team (2021). Initial lipid identification and annotation were carried out using LOBSTAHS (v 1.20; Collins (2016)), an open-source lipidomics annotation software based on ms1 spectra and adduct hierarchy. Putative annotations were verified, and isomeric/isobaric peaks with multiple annotations were deconvoluted with the *in silico* lipid fragmentation database in MS-Dial (v 4.80; Tsugawa (2020)). In addition, isomeric and isobaric annotations were verified by manual comparison of known(Popendorf et al., 2013) and observed MS2 fragment spectra of each compound.

Relative abundance of each peak area as calculated by XCMS and fed into as LOBSTAHS was normalized to the recovery of the PE peak in the internal standard to account for loss during extraction, variable response in ionization efficiency and ion suppression within the mass spectrometer. Based on the recovery of the different recovery internal standards, we confirmed that no one class of polar lipids was heavily impacted by ion suppression. After normalization, peak areas of the procedural blank were subtracted from all samples and compounds. Absolute lipid quantification was calculated using individual response factors of external standard curves. Standard curves were produced by running 0.625, 1.5, 2.5, and 5 ng of the EquiSPLASH standard on column using the aforementioned UHPLC-MS^n analytical method (Figure S5).

Pigments were annotated and quantified using standards of chlorophyll ɑ, chlorophyll *b*, astaxanthin, neoxanthin, violaxanthin purchased from Sigma-Aldrich (St Louis, MO, USA), and lutein, zeaxanthin purchased from Cayman Chemical Company (Ann Arbor, MI, USA). Putative pigment annotations were obtained based on the ms1 of the [M+H]+ adduct, as described in the default LOBSTAHS database(Collins et al., 2016). However, both the literature (Bijttebier et al., 2013; Juin et al., 2015; Milenković et al., 2012) and analysis of our pigment standards showed that dominant adducts vary between [M˙]+, [M+H]+, [M+Na]+ among different compounds, analytes, and analytical methods. We circumvented this uncertainty by summing the peak areas of [M˙]+, [M+H]+, [M+Na]+ adducts for each pigment compound. We could not distinguish between the isomers neoxanthin, prasinoxanthin, and

violaxanthin. Standard curves were produced with a series of concentrations: 0.0125, 0.025, 0.05, 0.1, 0.2 ng on column (Figure S2).

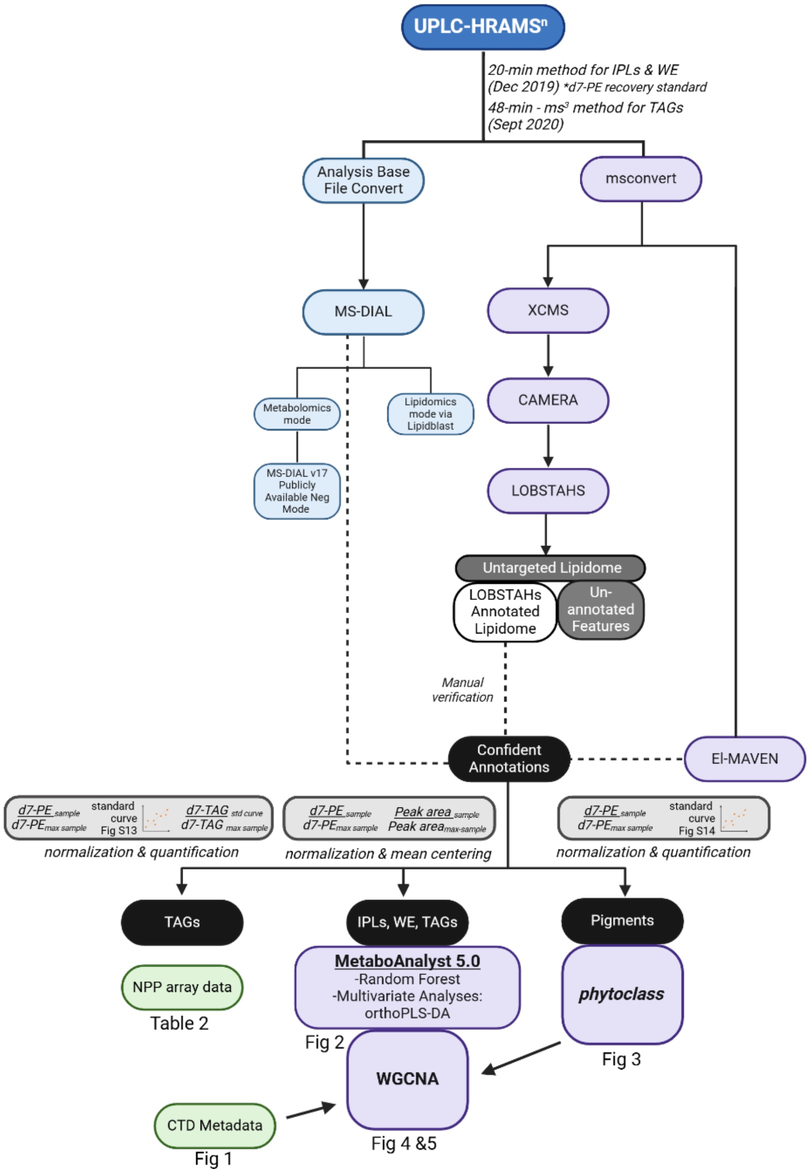

Figure S1. Lipidomic pipeline used to annotate and statistically analyze the CANON19 surface ocean lipidomes. The lipidomes were analyzed using ultra-high performance liquid chromatrography paired with high resolution accurate mass- msn mass spectrometry. The purple annotation route was followed using the pipeline set out in Collins *et al.* 2016 to give an initial annotation using the boutique database LOBSTAHS. The peak quality was manually check in the program El-MAVEN. The fragmentation was checked in MS-Dial (the turquoise route) using the LipidBlast database and the fragmentation for IPLs described in Popendorf *et al.* 2013. The resulting lipidome consisted of manually verified confident annotations. These peak areas were blank subtracted before normalizing for recovery of the internal standard. Pigments were quantified using a 5-point standard curve of five authentic standards (Fig S14) before using the R-package phytoclass to determine the phytoplankton community composition. The rest of the lipidome (IPLs, WE, TAGs, etc.) was analyzed in MetaboAnalyst using supervised routines for night vs. day and early vs. late, as well as in WGCNA to distinguish groups of lipids that had similar patterns in abundance over the 5-day time-series. Created in BioRender.

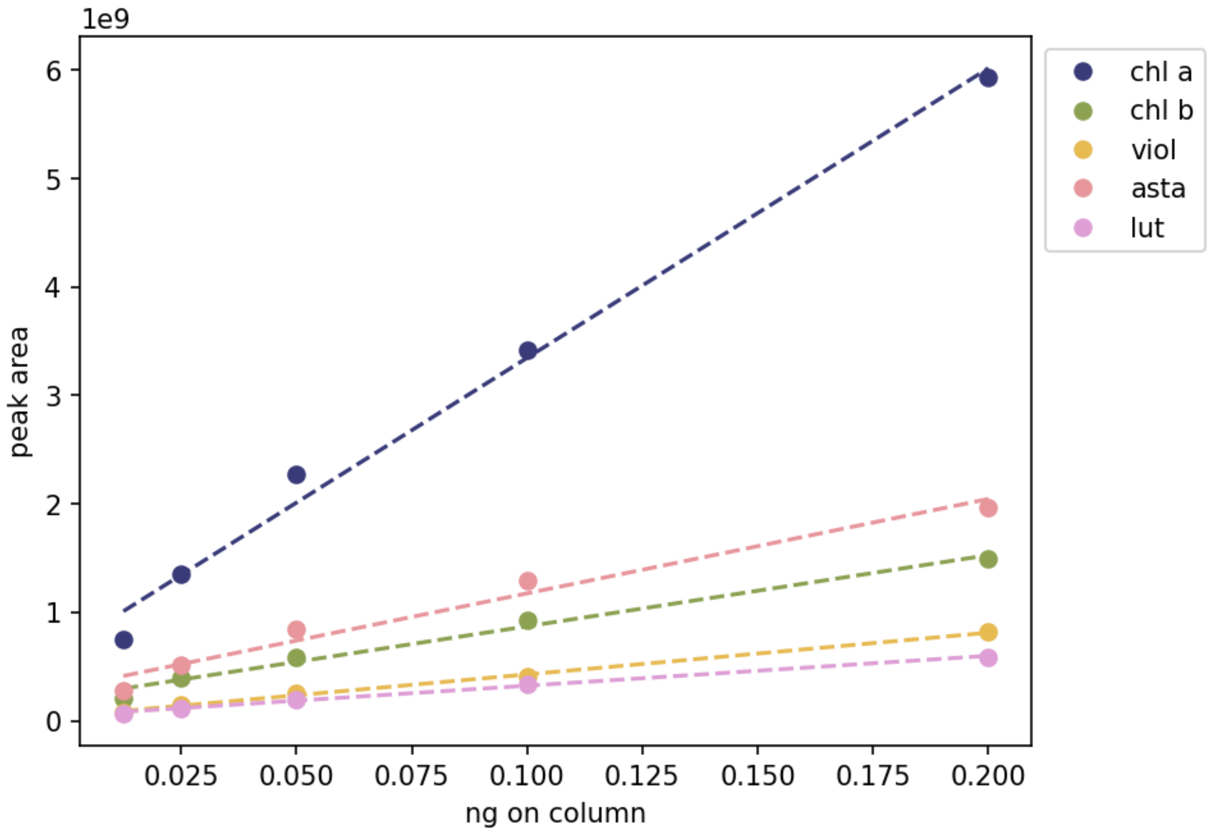

Figure S2. Standard curve of various pigment lipid standards (chl a & b: chlorophyll a & b, viol: violaxanthin, asta: astaxanthin, lut: lutein)

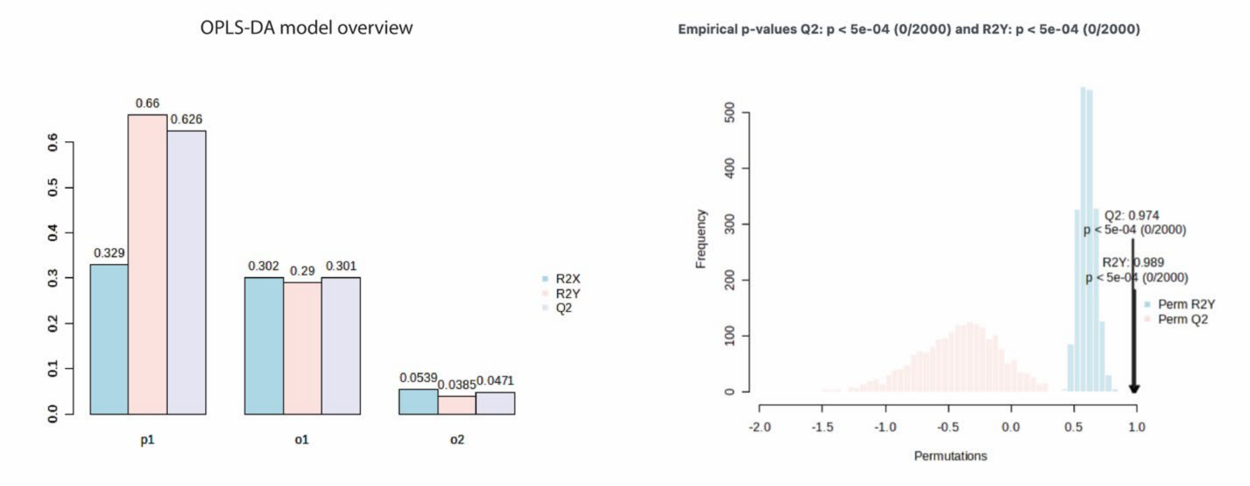

Figure S3. Model evaluation of OPLS-DA analysis presented in Figure 2.[1]

[1] [1] Szymańska, E., Saccenti, E., Smilde, A. K. & Westerhuis, J. A. Double-check: validation of diagnostic statistics for PLS-DA models in metabolomics studies. *Metabolomics* **8**, 3–16 (2012).

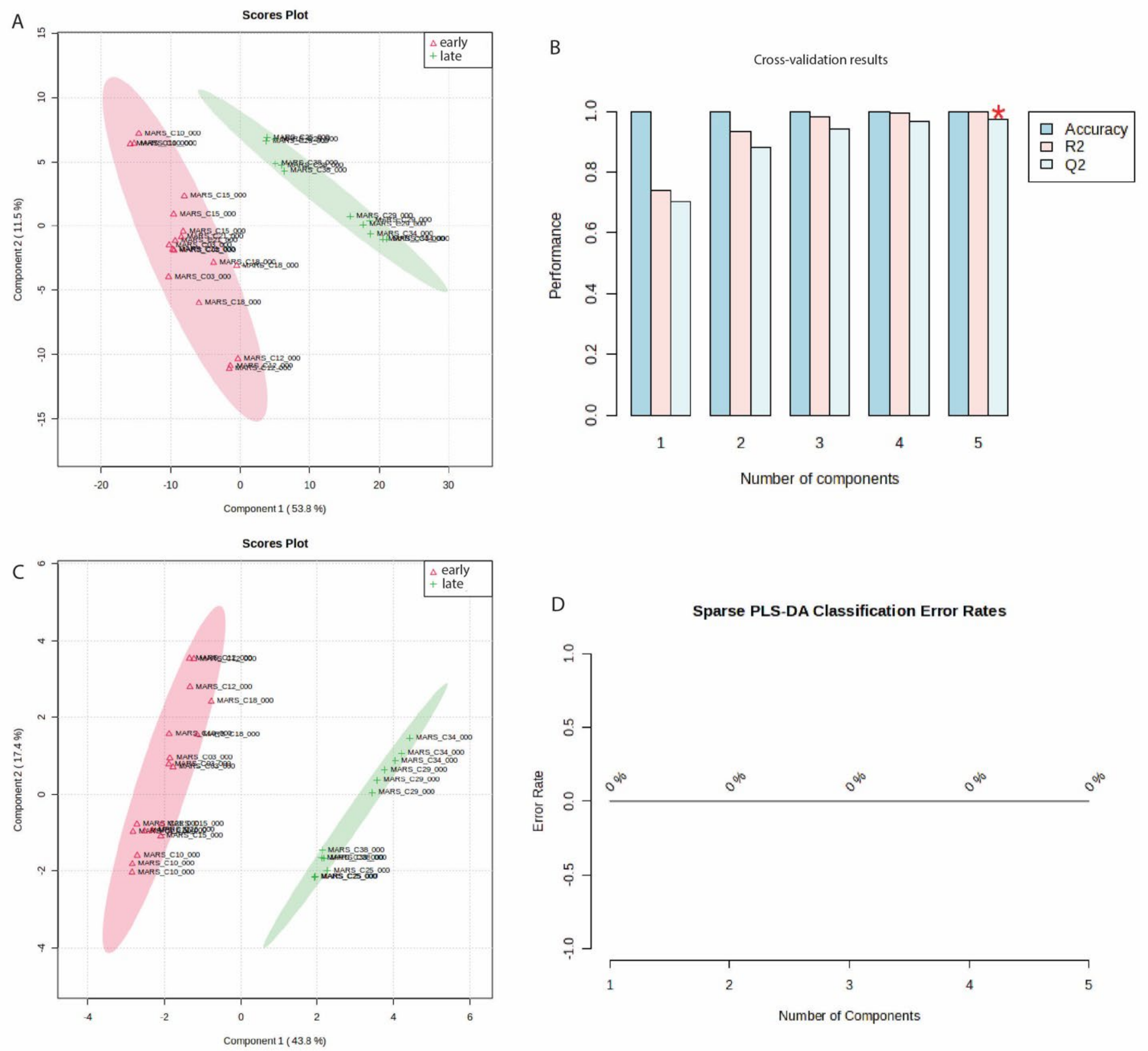

Figure S4. Multivariate analysis and model evaluation for samples categorized into early/late casts. (A) PLS-DA analysis results (B) 5-fold cross validation of PLS-DA model (C) sPLS-DA analysis results (D) 5-fold cross validation of sPLS-DA model

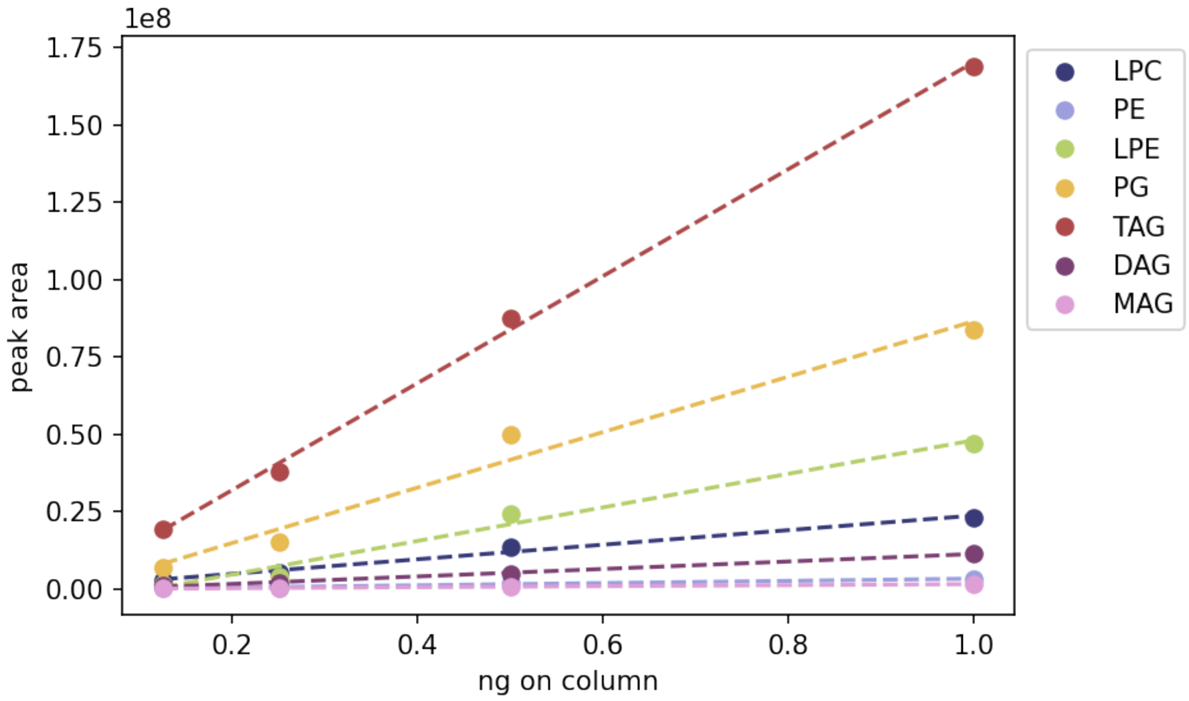

Figure S5. Standard curve of internal standard (EquiSPLASH LIPIDOMIX® Quantitative Mass Spec Internal Standard)

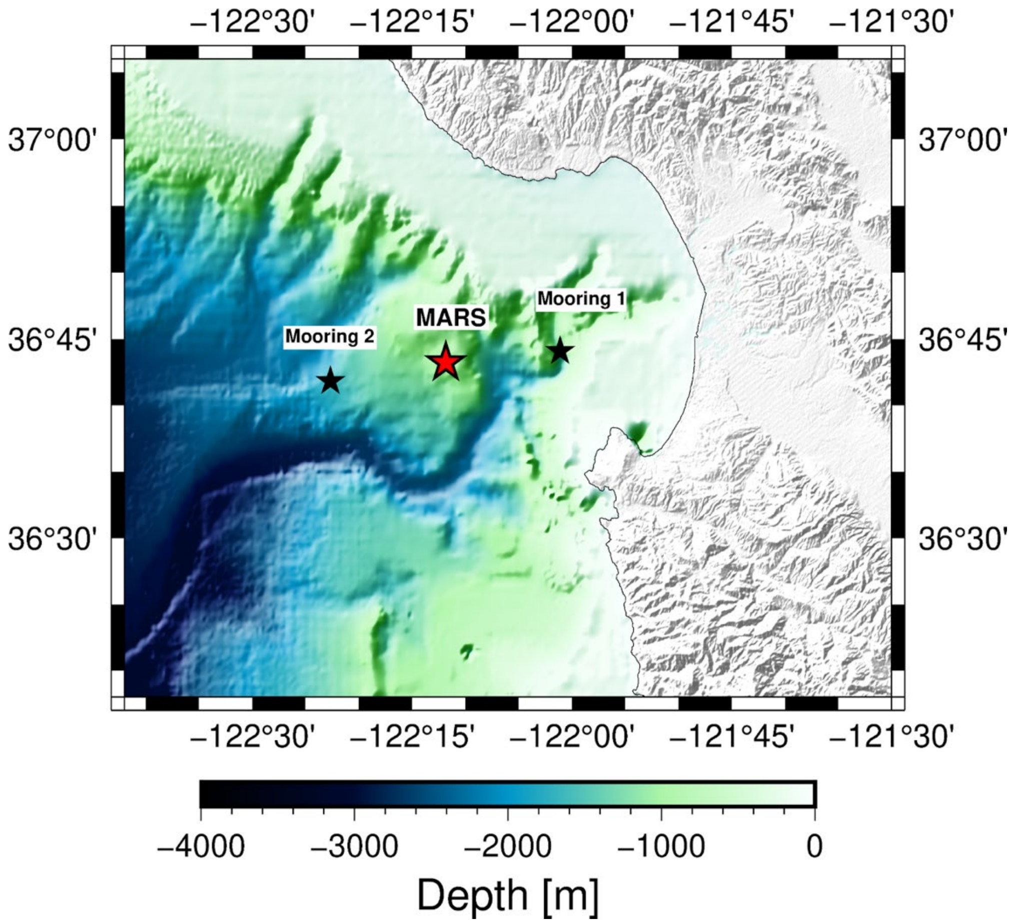

Figure S6. Map of moored sampling site in Monterey Bay (created with PyGMT)

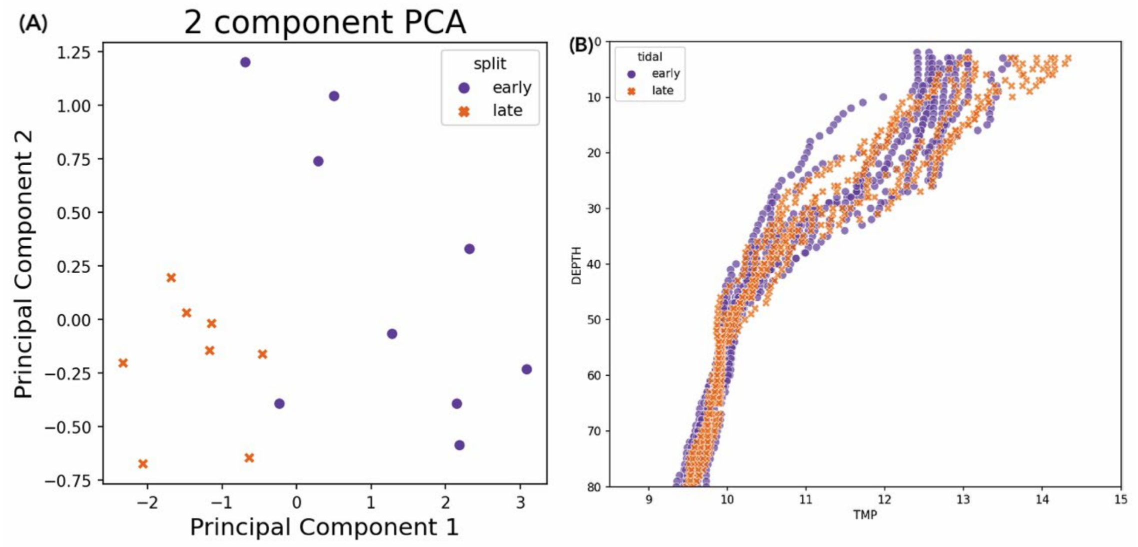

Figure S7. (A) 2 component PCA of ΔT, ΔS, ΔO2 between early (purple) and late (orange) period samples **(B)** Temperature-depth profile of early (purple) and late (orange) period samples

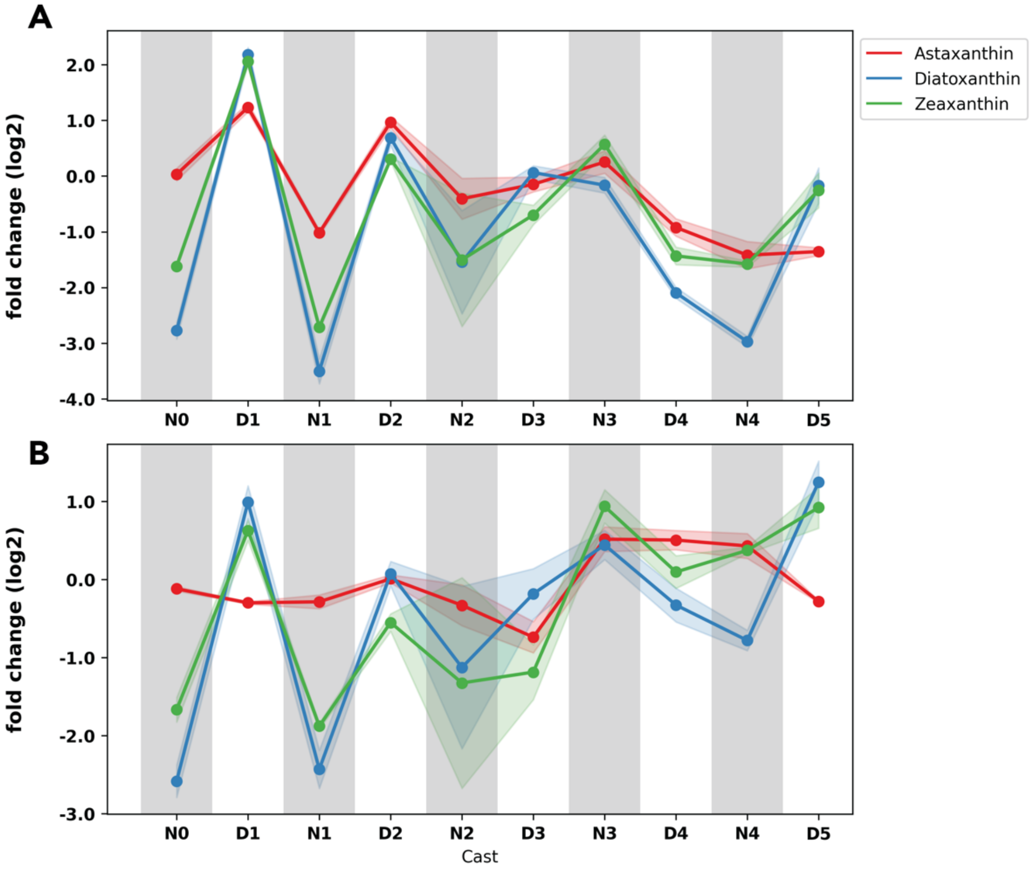

Figure S8. **(A)** Changes in pigments over time; y-axis is the normalized, mean-centered, and log_2-transformed peak area **(B)** Values of peak areas divided by the sum of the thylakoid glycolipids.

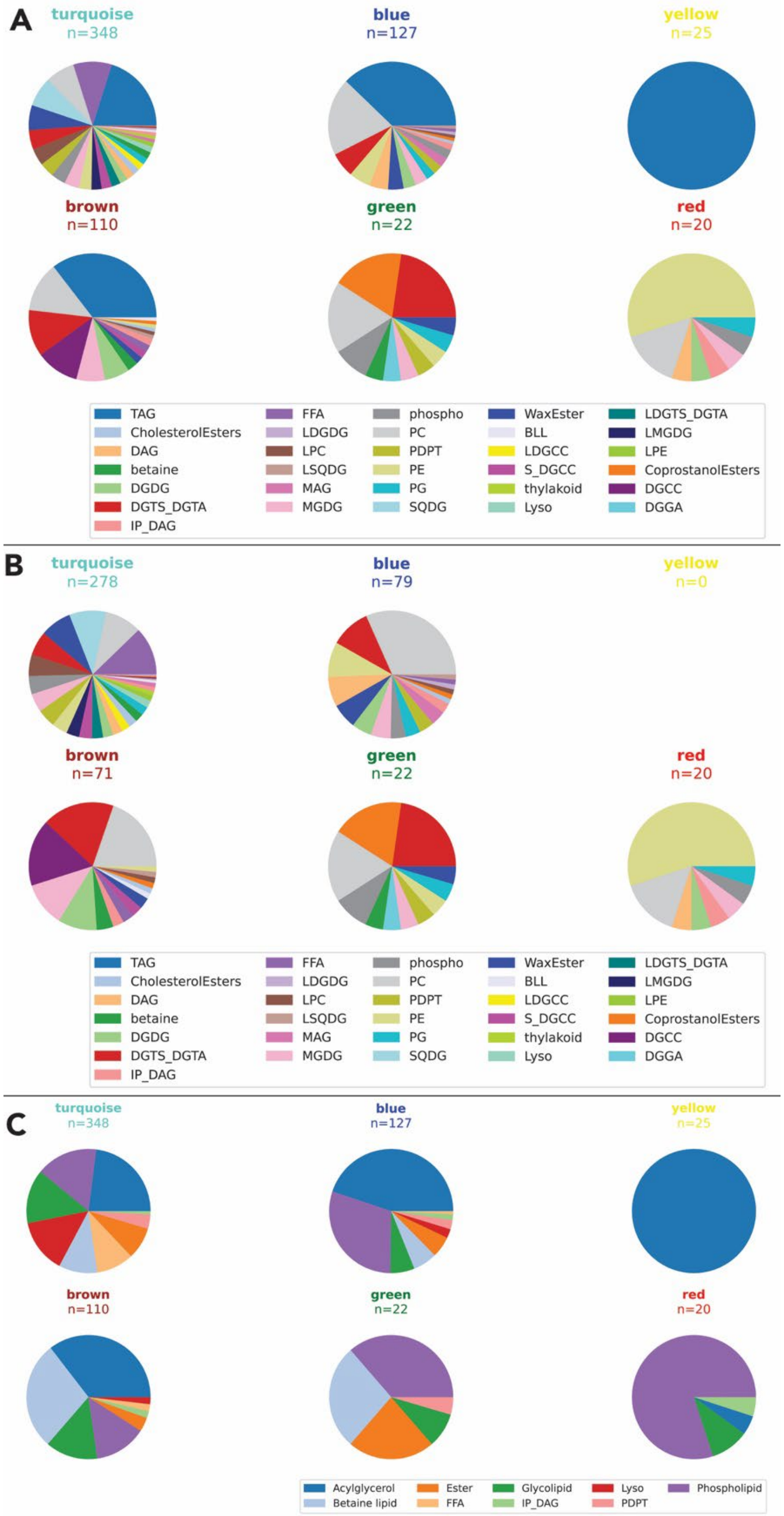

Figure S9. (A) Specific lipid class composition of WGCNA modules. **(B)** Specific non-TAG lipid class composition of WGCNA modules. The "betaine", "IP_DAG", "thylakoid", "Lyso", and "phospho" designate lipids of that class that had competing annotations in LOBSTAHS. **(C)** Lipid class composition clustered into groups based on their head groups

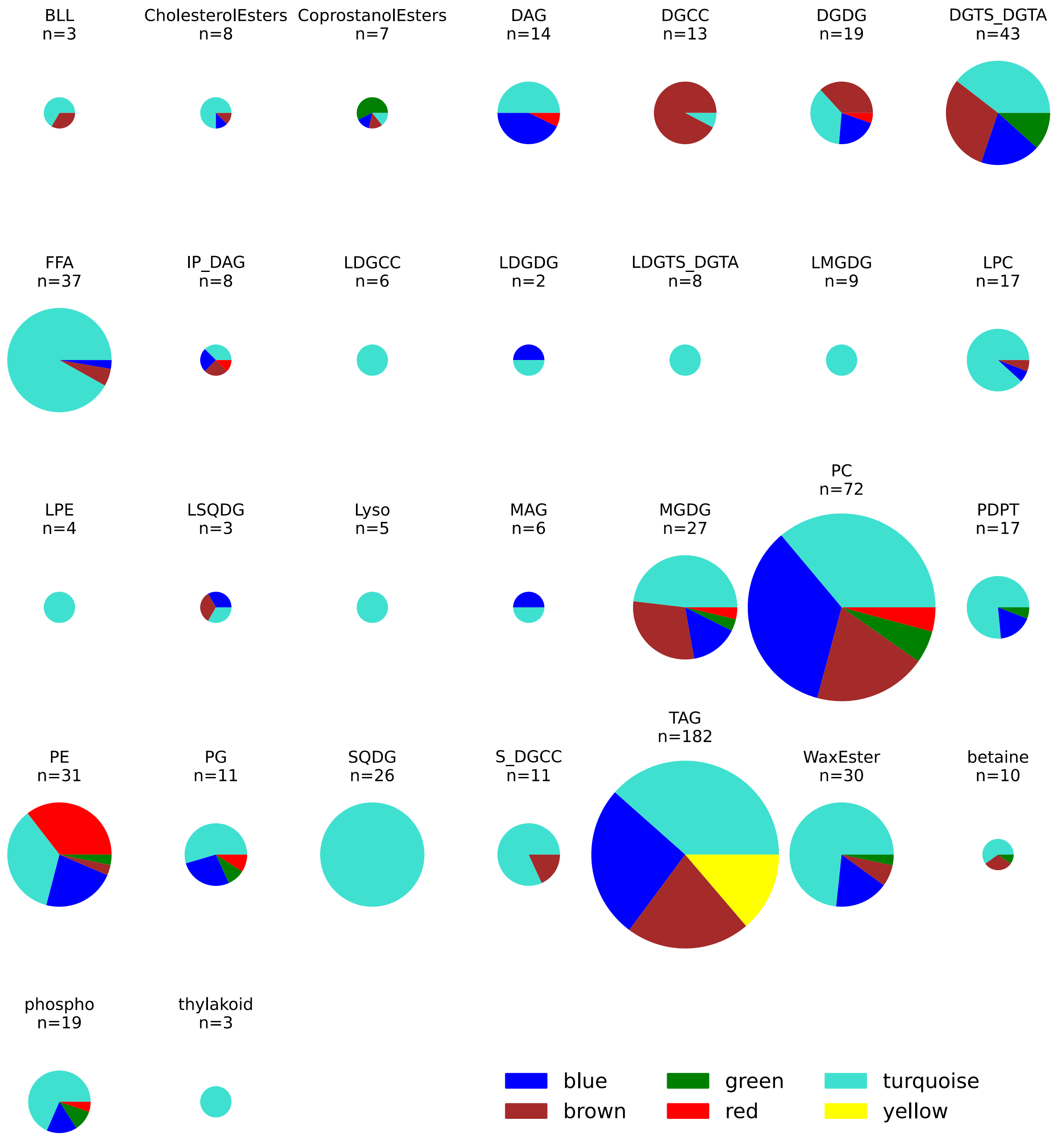

Figure S10. Module composition of each lipid class. Radii of pie charts are designated based on the number of components. The grey module, and lipids with competing annotations are not represented in the counts.

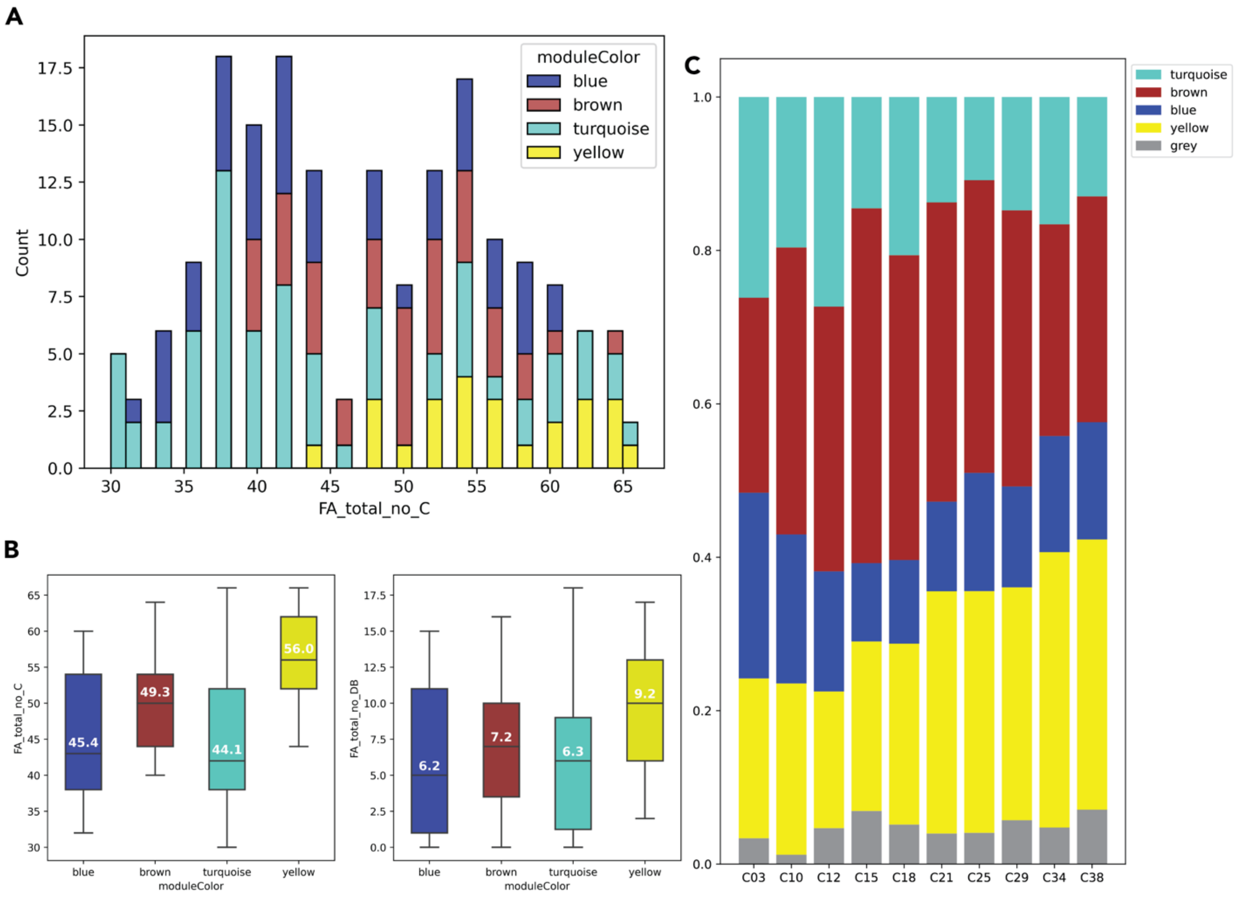

Figure S11. Composition of TAG modules based on (A) fatty acid chain lengths (stacked histogram) and (B) fatty acid chain lengths and number of double bonds (mean values are denoted in white) (C) TAG contribution of each module to TIC

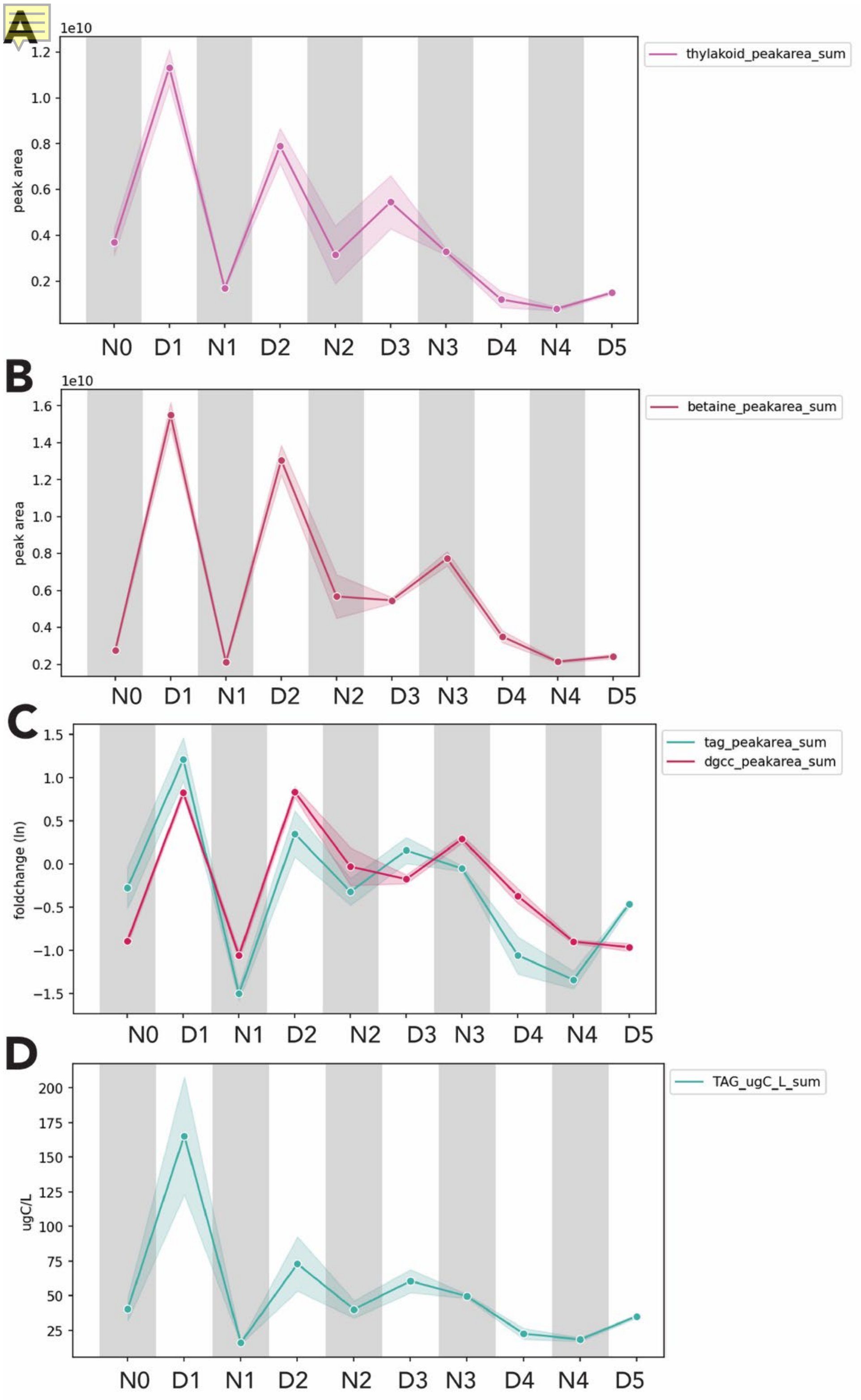

Figure S12. (A) Sum of annotated thylakoid glycolipids (MGDG, DGDG, SQDG; peak area) **(B)** Sum of annotated betaine lipids (DGCC, DGTS/DGTA, BLL; peak area) **(C)** Sum of annotated TAGs and DGCC (peak area) **(D)** Sum of quantified TAG-associated carbon (ugC of biomass per L), shading = 1σ across triplicates

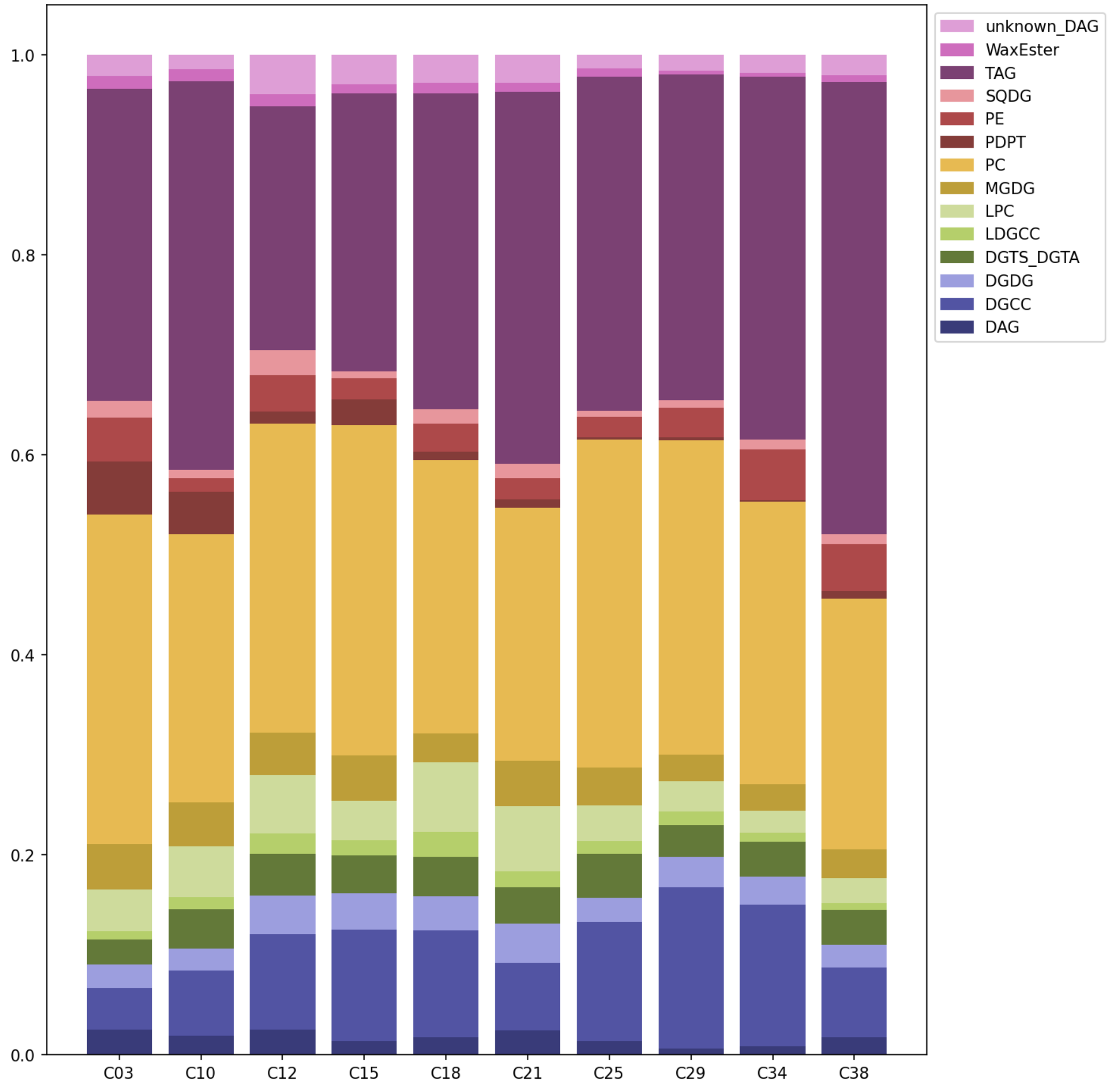

Figure S13. Relative contribution of lipid classes to total ion chromatogram (TIC). For clarity, lipid classes that contributed to less than 1% on average were removed. The "unknown_DAG" group designates intact polar lipids that had competing annotations in LOBSTAHS.

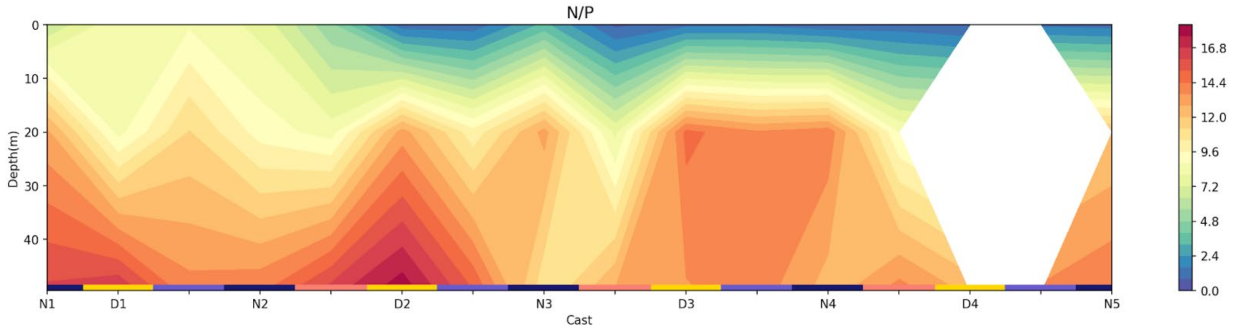

Figure S14. N:P ratio of surface (<50m) ocean. Nitrogen values are the sum of nitrate, nitrite, and ammonium measurements.

Table S1. OPLS-DA analysis scores for samples categorized into day/night casts

	All time points (N0-D5, n=30)	Early sampling phase (N0-D3, n=18)	Late sampling phase (N3-D5, n=12)
T-score (%)	7.4	**32.8**	10.5
Orthogonal T-score (%)	30.9	30.6	24.4

Table S2. Standard deviation of relative abundances of *phytoclass* phytoplankton groups

	Chlorophytes	Diatoms	Dinoflagellates	Haptophytes	Pelagophytes	*Synechococcus*
N0	0.090	0.101	0.151	0.055	0.000	0.000
D1	0.403	1.023	0.383	0.832	0.002	0.267
N1	0.456	0.489	0.103	0.085	0.000	0.177
D2	0.376	0.472	0.862	0.046	0.000	0.127
N2	7.026	19.716	8.604	10.120	0.019	1.740
D3	1.442	0.773	0.391	0.148	0.000	0.619
N3	2.352	4.800	1.219	2.261	0.036	4.087
D4	3.716	1.562	0.499	1.678	0.038	1.907
N4	1.238	4.009	0.752	1.059	0.061	3.011
D5	3.660	1.984	0.497	3.748	0.048	9.343

Table S3. Bakun Index at 36N x 122 W in units of m^3/s along 100 m of coastline from NOAA/NMFS/PFEG

Date	Bakun Index
2019-05-31	125
2019-06-01	75
2019-06-02	68
2019-06-03	111
2019-06-04	143

Table S4. Data Reduction Table for analysis of data via XCMS-CAMERA-LOBSTAHS-manual verification pipeline

	**Positive mode (20min method)**	**Negative mode**
**Initial peak groups determined with XCMS & CAMERA**	8195	8581
**Removal of isomers**	5797	5919
**Initial annotations**	7249	3593
**Removal of odd-chained FA**	4657	2336
**Application of Adduct Ion Hierarchy**	1615	786
**Manual verification of peaks**	1037	82

Table S5. Total and per-module TAG-associated carbon concentration over time-series

(μgC/mL)	N0	D1	N1	D2	N2	D3	N3	D4	N4	D5
Total	40.25	165.50	15.92	72.92	40.09	60.50	49.45	22.44	18.25	35.13
Turquoise	10.53	32.45	4.35	10.57	8.27	8.31	5.36	3.31	3.03	4.55
Yellow	8.39	36.98	2.84	16.12	9.45	19.10	15.58	6.81	6.55	12.38
Blue	9.74	32.15	2.50	7.45	4.37	7.07	7.62	2.95	2.76	5.37
Brown	10.23	61.91	5.50	33.72	15.92	23.60	18.87	8.08	5.04	10.34
Grey	1.35	2.02	0.75	5.05	2.07	2.42	2.02	1.28	0.87	2.49
